# Psychiatric and cognitive comorbidities of persistent post-traumatic headache attributed to mild traumatic brain injury

**DOI:** 10.1186/s10194-021-01287-7

**Published:** 2021-07-26

**Authors:** Håkan Ashina, Haidar Muhsen Al-Khazali, Afrim Iljazi, Sait Ashina, Faisal Mohammad Amin, Richard B. Lipton, Henrik Winther Schytz

**Affiliations:** 1 Department of Neurology, Danish Headache Center, Rigshospitalet Glostrup, Faculty of Health and Medical Sciences, University of Copenhagen, Valdemar Hansen Vej 5, DK-2600 Glostrup, Denmark; 2 Department of Neurorehabilitation and Traumatic Brain Injury, Rigshospitalet, Copenhagen, Denmark; 3 Comprehensive Headache Center, Beth Israel Deaconess Medical Center, Harvard Medical School, Boston, MA USA; 4 Department of Neurology, Beth Israel Deaconess Medical Center, Harvard Medical School, Boston, MA USA; 5 Department of Anesthesia, Critical Care and Pain Medicine, Beth Israel Deaconess Medical Center, Harvard Medical School, Boston, MA USA; 6 Department of Clinical Medicine, Faculty of Health and Medical Sciences, University of Copenhagen, Copenhagen, Denmark; 7 Department of Neurology, Albert Einstein College of Medicine, Bronx, NY USA; 8 Montefiore Headache Center, Bronx, NY USA

**Keywords:** Sleep, Anxiety, Depression, Cognitive impairment, Post-traumatic stress disorder

## Abstract

**Objective:**

To investigate the association of psychiatric and cognitive comorbidities with persistent post-traumatic headache (PTH) attributed to mild traumatic brain injury (TBI).

**Methods:**

A total of 100 patients with persistent PTH attributed to mild TBI and 100 age- and gender-matched healthy controls free of mild TBI were enrolled between July 2018 and June 2019. Quality of sleep was evaluated using the Pittsburgh Sleep Quality Index, while symptoms of anxiety and depression were assessed using the Hospital Anxiety and Depression Scale. Cognitive impairment was evaluated using the Montreal Cognitive Assessment questionnaire, while post-traumatic stress disorder (PTSD) was assessed using the Harvard Trauma Questionnaire.

**Results:**

In 100 patients with persistent PTH, 85% reported poor quality sleep, compared with 42% of healthy controls (*P* < 0.01). The relative frequency of probable to high risk of anxiety was 52% in the persistent PTH group vs. 8% in healthy controls (*P* < 0.01), while the relative frequency of probable to high risk of depression was 42% in the persistent PTH group vs. 2% in healthy controls (*P* < 0.01). Furthermore, 27% of the patients with persistent PTH had mild cognitive impairment while 10% had probable PTSD.

**Conclusions:**

Poor quality of sleep as well as symptoms suggestive of anxiety and depression were more common in patients with persistent PTH than healthy controls. Clinicians should screen patients with persistent PTH for these comorbidities and develop treatment plans that account for their presence.

## Introduction

Headache is a common sequela of mild traumatic brain injury (TBI), [1–3], as well as a frequent cause of chronic daily headache in the general population [4]. Few efforts have been made to assess the broader clinical picture of post-traumatic headache (PTH), [5–7]. The diagnosis of PTH is based on clinical criteria provided by the International Classification of Headache Disorders, 3rd edition (ICHD-3), and termed *headache attributed to traumatic injury to the head* [8]. Herein, PTH is characterized as either *acute* PTH, which develops within 7 days of the TBI and then remits within 3 months of onset, or *persistent* PTH, which persists beyond the 3 month-mark [8]. PTH is furthermore classified according to the severity of attributable TBI, being either mild or moderate to severe [8].

A key observation from clinic-based studies is that persistent PTH most often resembles a migraine-like headache phenotype, although some patients may report a ‘pure’ tension-type-like headache (TTH-like) phenotype [2, 5, 9]. Although the headache features of persistent PTH have been documented in multiple studies [2, 5, 6, 9], there is a scarcity of literature on associated comorbidities [7]. These include sleep disturbances, anxiety, depression, cognitive impairment, and post-traumatic stress disorder (PTSD) [7]. From a clinical standpoint, it is important to assess comorbidities as they are likely to have an important role in the long-term management and well-being of the patient. The presence of comorbidities might also affect the choice of therapy and, thus, facilitate more informed treatment approaches.

In this cross-sectional study, we assessed quality of sleep, anxiety, depression, cognitive impairment, and PTSD as comorbidities of persistent PTH. Age- and gender-matched healthy controls were used to compare rates of poor quality of sleep, anxiety, and depression. We hypothesized that symptoms suggestive of these comorbidities would be more frequent in patients with persistent PTH, compared with healthy controls.

## Methods

### Study population

We enrolled patients with persistent PTH attributed to mild TBI. Patients were recruited from the outpatient clinic of the Danish Headache Center, the Danish post-concussion syndrome support group website (hjernerystelsesforeningen.dk), and from neurological departments and rehabilitation centers in the Capital Region of Denmark. Healthy non-headache controls were recruited through the Danish research subject website (http://gsperson.dk) and posters placed at various public institutions in the Capital Region of Denmark. A trained locum doctor (HMA) was responsible for the initial screening of study eligibility by phone.

This article is part of a larger parental study that was approved by the Regional Health Research Ethics Committee of the Capital Region of Denmark (H-18011477), [5]. All participants gave written consent after receiving detailed oral and written information. The study was conducted at the Danish Headache Center in accordance with the Declaration of Helsinki [10], with later revisions. The study was also approved by the Danish Data Protection Agency.

### Inclusion and exclusion criteria

The diagnosis of persistent PTH attributed to mild TBI was established by a trained locum doctor (AI) using a semi-structured interview and made in accordance with the ICHD-3 criteria for *persistent headache attributed to mild traumatic injury to the head* [8]. Table 1 sets out the ICHD-3 criteria for mild traumatic injury to the head. Other inclusion criteria for individuals with persistent PTH were: 1) mild TBI to have occurred at least 12 months prior to study participation and 2) age between 18 and 65 years. Exclusion criteria were 1) any history of primary headache disorder (except infrequent TTH), 2) any history of whiplash injury or more than one TBI, 3) pregnant or nursing women, 4) cardiovascular or cerebrovascular disease, 5) pre-trauma psychiatric disorder (unless well-regulated), and 6) medication-overuse headache. To ensure eligibility for inclusion, medical records were reviewed to cross-check for any formal medical or psychiatric diagnosis. Moreover, we required no intake of analgesics within 24 h of study participation because study participants were scheduled for functional magnetic resonance imaging and blood sampling as part of the larger parental study. Some of the data from the larger parental study have been published elsewhere [5, 11, 12].

**Table 1:** Diagnostic criteria for mild traumatic injury to the head.^a^

Mild traumatic injury to the head	
Injury to the head fulfilling both of the following:	
1. Associated with none of the following:	
Loss of consciousness for > 30 min	
Glasgow Coma Scale (GCS) score < 13	
Post-traumatic amnesia lasting > 24 h1	
Altered level of awareness for > 24 h	
Imaging evidence of a traumatic head injury such as skull fracture, intracranial haemorrhage and/or brain contusion	
2. Associated with one or more of the following symptoms and/or signs	
transient confusion, disorientation or impaired consciousness	
loss of memory for events immediately before or after the head injury	
two or more of the following symptoms suggestive of mild traumatic brain injury:	
nausea	
vomiting	
visual disturbances	
dizziness and/or vertigo	
gait and/or postural imbalance	
impaired memory and/or concentration	

^a^Diagnostic criteria are from the International Classification of Headache Disorders, 3rd edition (ICHD-3), [8]

Inclusion criteria for healthy controls were 1) age between 18 and 65 years, 2) no history of known head trauma or whiplash injury, 3) no history of primary headache disorder (except infrequent episodic TTH), 4) no first-degree relatives with primary headache disorder, 5) no daily intake of medicine other than oral contraceptives, 6) no history of neurological or psychological disorders, 7) no history of structural heart disease. An in-person structured interview was performed by a trained locum doctor (AI) to determine study eligibility. Medical records were reviewed to cross-check for any formal medical or psychiatric diagnosis.

### Measures

An in-person semi-structured interview was used to record data on demographics, medical history, and full clinical course. The assignment of headache phenotypes was based on review of clinical features in patients with persistent PTH and without consideration of the ICHD-3 criterion for primary headache disorders, which states “not better accounted for by another ICHD-3 diagnosis”. As study inclusion was based on a diagnosis of persistent PTH attributed mild TBI, we used terms such as migraine-like and TTH-like to describe assigned headache phenotypes. The case definitions, that were used to classify migraine-like and TTH-like days, have been published elsewhere [13].

### Pittsburg sleep quality index

The Pittsburg Sleep Quality Index (PSQ-I) is a 19-item self-report instrument that comprises 7 components used to assess quality of sleep [14]. Each component is scored on a scale of 0 to 3, which is then used to calculate a global score of 0 to 21. Poor quality of sleep is defined as global scores of 5 or higher. The appraisal period is the past 30 days.

### Hospital anxiety and depression scale

The Hospital Anxiety and Depression Scale (HADS) is a 14-item self-report instrument used to screen for anxiety and depression separately [15]. The range of the scale is 0 to 21, with scores of 8 to 10 being indicative of a probable risk of anxiety or depression, whereas scores of 11 to 21 indicate a high risk of anxiety or depression. The appraisal period is the past 7 days.

### Montreal cognitive assessment

The Montreal Cognitive Assessment (MoCA) questionnaire is a 30-item instrument used to screen for cognitive impairment [16]. The range of the scale is 0 to 30, and scores of 26 or less are indicative of cognitive impairment. The degree of cognitive impairment is denoted as mild if scores are between 18 to 25. The MoCA questionnaire was only administered to patients with persistent PTH.

### Harvard trauma questionnaire

The Harvard Trauma Questionnaire (HTQ) is a 16-item instrument used to screen for PTSD [17]. Each item is rated on a four-point Likert scale, and total mean scores of 2.5 or higher are indicative of probable PTSD. The appraisal period is the past 7 days. The HTQ questionnaire was only administered to patients with persistent PTH.

### Statistical analysis

Descriptive statistics were used to summarize baseline characteristics. Continuous variable were reported as means and standard deviations (SDs), whereas categorical variables were summarized as proportions and/or percentages. Comparisons were performed using a *t* test for continuous variables and a χ^2^ test for categorical variables. For analyses of correlation, we used the Spearman rank correlation coefficient (r_s_). All statistical analyses were conducted using IBM SPSS Statistics for Windows, Version 25.0, Armonk, NY, USA.

## Results

A total of 100 patients with persistent PTH and 100 age- and gender-matched healthy controls were enrolled between July 2018 and June 2019. Of 100 participants in both groups, 83 were females and 17 were males. The mean age (SD) was 36.0 (11.6) years in patients with persistent PTH and 35.8 (11.3) years in healthy controls. Furthermore, the mean BMI (SD) was 24.5 (4.1) kg/m^2^ in patients with persistent PTH and 24.3 (3.7) kg/m^2^ in healthy controls. A more detailed summary has been published elsewhere [5] and is also presented in Tables 2, 3 and 4. In brief, patients with persistent PTH had a mean (SD) headache frequency of 25.4 (7.1) days per month. The most common headache phenotypes were chronic migraine-like (*n* = 61) followed by combined episodic migraine-like and episodic/chronic TTH-like (*n* = 29), ‘pure’ chronic TTH-like (*n* = 9), and episodic migraine-like headache (*n* = 1), (Table 3).

**Table 2:** Summary Characteristics

Variable	Persistent PTH (***n*** = 100)	Healthy Controls (***n*** = 100)
**Age**, mean (SD), y	36.0 (11.7)	35.8 (11.3)
**Male/Female**, %	17/83	18/82
**Height**, mean (SD), cm	171.3 (8.2)	170.9 (8.7)
**Weight**, mean (SD), kg	72.1 (14.4)	71.2 (14.2)
**Body Mass Index,** mean (SD), kg/m^2^	24.5 (4.1)	24.3 (3.7)

**Table 3:** Characteristics of Patients with Persistent Post-Traumatic Headache

Variable	Persistent PTH (***n*** = 100)
**Age**, mean (SD), y	36.0 (11.7)
**Male/Female**, %	17/83
**Height**, mean (SD), cm	171.3 (8.2)
**Weight**, mean (SD), kg	72.1 (14.4)
**Body Mass Index,** mean (SD), kg/m^2^	24.5 (4.1)
**Current Employment Status**, %	
Full-time employed	37
Part-time employed	42
Unemployed	21
**Education**	
Years of education, mean (SD), y	15.6 (2.8)
No education besides completion of secondary school or high school, %	17
Skilled labor, %	25
Bachelor’s degree, %	38
Higher education, %	20
**Injury Cause**, %	
Fall	42
Motor vehicle collision	18
Sports-related injury	8
Violence/assault	2
Other unintentional injury	30
**Localization of Mild TBI**, %	
Frontal	41
Temporal	27
Parietal	15
Occipital	27
**Mild TBI associated with**, %	
Transient confusion, disorientation, or impaired consciousness	82
Loss of memory for events immediately before or after the head injury	67
Nausea	82
Vomiting	24
Visual disturbances	46
Dizziness and/or vertigo	87
Gait and/or postural imbalance	53
Impaired memory and/or concentration	84
**Months with Headache attributed to Mild TBI**, mean (SD), months	49.3 (37.3)
**Headache Phenotypes**, %	
Chronic migraine-like	61
Episodic migraine-like	1
Episodic migraine-like combined with chronic TTH-like	25
Episodic migraine-like combined with frequent TTH-like	2
Episodic migraine-like combined with infrequent TTH-like	2
Chronic TTH-like	9
**Self-Rated Health**, %	
Excellent	2
Great	12
Good	34
Rather poor	38
Poor	14
**Medico-Legal Issues / Litigation**, %	
Ongoing litigation	31
Ended litigation	44
Improvement in headache following end of litigation, No. of subjects (%)	1 (2.3%)
**Pre-Trauma Psychiatric History**, %	23
Anxiety disorders	12
Major depressive disorder	14
Eating disorders	4
**Pre-Trauma Chronic Pain Conditions**^**a**^, %	11
Neck pain	0
Low back pain	3
Neuropathic pain	1
Other chronic pain conditions	9

^a^chronic pain was defined as persistent or recurrent pain that lasted longer than 3 months

**Table 4:** Headache Characteristics of the Study Population

Variable	Persistent PTH (*n* = 100)	Chronic Migraine-Like (*n* = 61)	Episodic Migraine-Like combined with TTH-like (*n* = 29)	Chronic TTH-like (*n* = 9)
**Headache Frequency**, mean (SD)				
Yearly headache days	307.9 (86.9)	299.3 (88.3)	318.5 (83.0)	358.1 (12.8)
Monthly headache days	25.4 (7.1)	24.6 (7.5)	26.2 (6.8)	29.7 (0.7)
**Headache Intensity**^**a**^, %				
Mild	5	0	0	56
Moderate	80	82	86	44
Severe	15	18	14	0
**Headache Localization**, %				
Bilateral	64	69	62	56
Unilateral	36	31	38	44
Left sided	18	16	17	22
Right sided	17	15	21	22
Frontal	70	70	72	67
Temporal	50	51	41	67
Parietal	35	39	21	44
Occipital	35	41	24	33
**Headache Quality**, %				
Throbbing	18	23	14	0
Pressing	32	21	31	100
Stabbing	3	2	7	0
Combined (throbbing and pressing)	45	51	48	0
Other headache quality	2	3	0	0
**Family History of Primary Headache Disorders**^**b**^, %	28	31	21	22
**Continuous Photophobia**^**c**^, %	46	69	45	44
**Continuous Phonophobia**^**d**^, %	60	61	28	0
**Neck Pain**, %	78	82	79	44
> 180 days with neck pain within the past 12 months, %	55	78	52	33
**Low Back Pain**, %	37	38	45	11
> 180 days with neck pain within the past 12 months, %	24	26	23	0
**Allodynia**^**e**^				
None, %	54	44	66	78
Mild Allodynia, %	23	25	21	22
Moderate Allodynia, %	17	23	10	0
Severe Allodynia, %	6	8	3	0

^a^*mild* headache intensity = does not impair ability to work and/or other activities. *Moderate* headache intensity = impairs but does not prevent ability to work and/or other activities. *Severe* headache intensity = prevents ability to work and/or other activities

^b^positive family history of primary headache disorders = 1st degree relative with any primary headache disorder other than infrequent tension-type headache

^c^continuous photophobia = continuously ongoing – daily – symptoms of photophobia

^d^continuous phonophobia = continuously ongoing – daily – symptoms of phonophobia

^e^cutaneous allodynia scores were assessed using the 12-item Allodynia Symptom Checklist (ASC-12) and defined as follows: *none* (scores 0–2), *mild* [3–5], *moderate* [6–8], and *severe* (≥9)

### Associated comorbidities

Tables 5 and 6 summarize outcome rates for poor quality of sleep, anxiety, depression, mild cognitive impairment, and PTSD. In 100 patients with persistent PTH, 85% had poor quality of sleep, 52% had at least probable risk of anxiety, 42% had at least probable risk of depression, 27% had mild cognitive impairment, and 10% had probable PTSD. Compared with healthy controls, patients with persistent PTH had a higher relative frequency of poor quality of sleep (85% vs. 42%, *P* < 0.01), anxiety (52% vs. 8%, *P* < 0.01), and depression (42% vs. 2%, *P* < 0.01). Another observation was that 28% of patients with persistent PTH had poor quality of sleep, as well as at least probable risk of both anxiety and depression.

**Table 5:** Comorbidities of the Study Population

Comorbidities	Persistent PTH (*n* = 100)	Healthy Controls (*n* = 100)	*P* value
**Subjective Sleep Quality**			
Poor Sleep Quality^a^, No.	85	42	< 0.01
**Anxiety**			< 0.01
Probable or High Risk of Anxiety, No.	52	8	
Probable Risk of Anxiety, %	19	8	
High Risk of Anxiety, %	33	0	
**Depression**			< 0.01
Probable or High Risk of Depression, No.	42	2	
Probable Risk of Depression, %	30	2	
High Risk of Depression, %	12	0	
**Mild Cognitive Impairment**, No.	27	N/A	N/A
**Post-Traumatic Stress Disorder**, No.	10	N/A	N/A

**Table 6:** Comorbidities of the Chronic Migraine-Like Group

Comorbidities	Chronic Migraine-Like (*n* = 61)	Other Headache Phenotypes (*n* = 39)	*P* value
**Subjective Sleep Quality**			
Poor Sleep Quality, %	93	72	< 0.05
**Anxiety**			
Probable or High Risk of Anxiety, %	46	54	0.35
**Depression**			
Probable or High Risk of Depression, %	56	74	0.32

In 91 patients with a migraine-like phenotype, 88% had poor quality of sleep, 56% had at least probable risk of anxiety, and 43% had at least probable risk of depression. The corresponding figures for 61 patients with a chronic migraine-like phenotype were 93%, 46%, and 56% (Table 6).

### Correlations

Global PSQ-I scores correlated with HADS anxiety scores (*r* = 0.31; *P* < 0.01) and HADS depression scores (*r* = 0.37; *P* < 0.01), whereas no significant correlation was found with monthly headache days (*r* = 0.19; *P* = 0.18), monthly migraine-like days (*r* = 0.19; *P* = 0.08), or months with headache attributed to mild TBI (*r* = 0.08; *P* = 0.42). HADS anxiety scores correlated with HADS depression scores (*r* = 0.56; *P* < 0.01), whilst no relationship was observed with monthly headache days (*r* = − 0.02; *P* = 0.86), monthly migraine days (*r* = 0.02; *P* = 0.83), or months with headache attributed to mild TBI (*r* = − 0.13; *P* = 0.19). HADS depression scores did not correlate with monthly headache days (*r* = 0.02; *P* = 0.84), monthly migraine days (*r* = 0.15; *P* = 0.15), or months with headache attributed to mild TBI (*r* = − 12; *P* = 0.23).

## Discussion

This study presents data on comorbidities in 100 patients with persistent PTH and 100 age- and gender-matched healthy controls. When comparing the two groups, poor quality of sleep (85% vs. 42%), at least probable risk of anxiety (52% vs. 8%), and at least probable risk of depression (42% vs. 2%) were more frequently observed in patients with persistent PTH. In addition, mild cognitive impairment was present in 27% of patients with persistent PTH, whilst probable PTSD was found in 10%. These findings underscore that patients with persistent PTH often experience symptoms suggestive of psychiatric and cognitive comorbidities. An initial screening for comorbid conditions should be used in clinical practice to facilitate treatment schemes that are more carefully matched to patients. This, in turn, would be a much needed move away from the conventional one-size-fits-all approach.

Our finding of poor quality of sleep in most patients with persistent PTH is in accord with previous observations [7, 18]. A recent clinic-based study found that 26% of patients with persistent PTH had a severe degree of insomnia, whilst 40% had a moderate degree of insomnia [18]. The same study also found that insomnia was more frequent in patients with persistent PTH, compared with patients with migraine [18]. This is an interesting observation because a recent web-based survey reported that insomnia is more common in individuals with migraine than non-migraine controls [19]. A meta-analysis has estimated that sleep disturbances are experienced by about 50% of individuals who sustain a TBI [20], Taken together, it is evident that poor quality of sleep is prevalent in patients with persistent PTH attributed to mild TBI.

Besides poor quality of sleep, we found that anxiety and depression are more frequent in patients with persistent PTH, compared with healthy controls. These associations have several possible explanations. For example, anxiety and depression may be independent risk factors for the development of TBI-related sequelae, such as persistent PTH. Another option is that anxiety and depression are sequelae of TBI that are unrelated to persistent PTH per se. Lastly, they could indeed be linked to persistent PTH. Many studies indicate that that anxiety and depression are common sequela in patients with TBI [21, 22]. A prospective cohort study found that the relative frequency of anxiety and depression was higher in patients with persistent PTH, compared with those who had sustained a mild TBI but did not report headache [23]. Longitudinal data is required to resolve the timing of PTH, anxiety, depression following mild TBI.

An interesting finding from our study is that mild cognitive impairment was experienced by 27% of patients with persistent PTH. This is in line with findings from a prospective cohort study of patients with mild TBI [24]. The authors reported that about 60% of patients, who had sustained a mild TBI, reported at least one cognitive symptom at 6 months and 12 months post-trauma [24]. Furthermore, another study found that cognitive performance at 2 weeks post-injury was lower in patients with mild TBI when compared with healthy controls [25].

Although there are robust data that the risk of PTSD is increased after mild TBI [22, 26, 27], it remains unclear to what extent individuals with persistent PTH are affected by PTSD. Two small clinic-based studies have found that about 30% of patients with persistent PTH have PTSD [28, 29]. In contrast, we found that 10% of patients with persistent PTH had probable PTSD. The discordant findings could be due to differences in methodology or the pre-injury characteristics of the study populations. Nonetheless, it should be noted that some experimental data does support a possible bidirectional relation between PTH and PTSD, possibly attributed to dysfunction in pain modulatory circuits [30].

### Limitations

Several limitations should be noted before considering the implications of our findings. First, we excluded individuals with a personal history of primary headache disorder (except infrequent episodic TTH) from inclusion in the control group [8]. This might have affected the observed relative frequencies of poor quality of sleep, anxiety, and depression. Indeed, there is evidence to suggest an association of migraine and TTH with these comorbidities [19, 31–33]. Another point, that merits emphasis, is the occurrence of pre-trauma psychiatric history in 23% of patient with persistent PTH, compared with none in the control group. This might yield higher comorbidity scores for patients with persistent PTH. However, it should be mentioned that none of the patients with persistent PTH received treatment for any psychiatric disorder at the time of the mild TBI. Second, we used questionnaires for screening of comorbidities rather than diagnostic instruments. This could result in an over- or underestimation of the caseness prevalence. In addition, we are not able to provide a detailed clinical description of the assessed comorbidities when using screeners. Third, we used a cross-sectional design, which therefore does not allow for causality to be inferred. This study should, therefore, be considered a first step in the investigation of comorbidities associated with persistent PTH. Causation should be ascertained in future prospective cohort studies. Lastly, we recruited patients with persistent PTH who had sought medical care or were involved with patient-support groups. Thus, it is plausible that our study population is skewed towards more adversely affected individuals with persistent PTH.

## Conclusions

Poor quality of sleep, anxiety, and depression are more common in patients with persistent PTH, compared with healthy controls. In addition, some patients with persistent PTH have symptoms suggestive of mild cognitive impairment and PTSD. These findings underscore that patients should be screened for common comorbidities during the initial clinical assessment. Recognition of these might, in turn, affect choice of therapy and highlight the need for more active follow-up. In addition, effective management of comorbidities might be useful in improving treatment outcomes for persistent PTH.

## Data Availability

Qualified researchers can request access to patient-level data and related study documents, including the study protocol. Patient-level data will be deidentified and study documents will be redacted to protect the privacy of study participants.

## Abbreviations

TBI: Traumatic brain injury
PTH: Post-traumatic headache
ICHD-3: International Classification of Headache Disorders, Third Edition
TTH-like: Tension-type headache-like
PTSD: Post-traumatic stress disorder
PSQ-I: Pittsburg Sleep Quality Index
HADS: Hospital Anxiety and Depression Scale
MoCA: Montreal Cognitive Assessment
HTQ: Harvard Trauma Questionnaire
